# Tumor Methylation Burden (TMeB) in Non-Small Cell Lung Cancer: A New Way of Thinking About Epigenetics

**DOI:** 10.3390/ijms252312966

**Published:** 2024-12-02

**Authors:** Federico Pio Fabrizio, Lucia Anna Muscarella

**Affiliations:** 1 Faculty of Medicine and Surgery, “Kore” University of Enna, 94100 Enna, Italy; 2 Laboratory of Oncology, IRCCS Casa Sollievo della Sofferenza, 71013 San Giovanni Rotondo, Italy; l.muscarella@operapadrepio.it

Lung cancer represents a substantial proportion of cancer-associated mortality worldwide, with non-small cell lung cancer (NSCLC) accounting for most of these cases [1]. Despite the significant progress in targeted therapies and immunotherapy as effective and promising therapeutic options for lung cancer patients at all disease stages, most of the time, lung cancer survival remains scarce due to resistance, relapse, or minimal response to treatments [2]. Consequently, one of the main challenges is to detect lung cancer at early stages using multiple technical approaches and combining multiple molecular markers in order to reduce mortality rates [3].

In the last ten years, research in the epigenetic field has been rapidly developing, with DNA methylation becoming the most “genetic” of the epigenetic hallmarks in cancer, due to its stable features and location close to the “naked” DNA sequences. Methylation changes in cancer mainly consist of an addition of a methyl group (CH_3_) from S-adenosyl-L-methionine to the C-5 position of a cytosine residue to form 5-methylcytosine (5mC), catalyzed by DNA methyltransferases (DNMTs) [4]. In mammals, DNA methylation suppresses gene expression and is involved in normal cellular processes, as well as appearing to be deregulated in tumor progression, metastatic spread, and therapy resistance in many cancer types, such as breast, colon, and lung cancer [5,6].

Technically, strand-specific CpG methylation profiling in targeted regions of the selected genes could be detectable using well-known standard methods or high-throughput assays based either on microarrays (e.g., Infinium HumanMethylation450 BeadChip, MethylationEPIC BeadChip; Illumina, San Diego, CA, USA) or next-generation sequencing (e.g., Custom Targeted NGS Panels, reduced representation bisulfite sequencing, RRBS), thus facilitating the identification of complex epigenetic patterns specific to cancer cells [7,8,9]. In many labs, the ability to overcome pre-analytical and analytical criticisms related to the DNA fragmentation in formalin-fixed paraffin-embedded (FFPE) lung cancer tissues to improve bisulfite sequencing on high-throughput platforms is rapidly becoming a practical reality. Therefore, the definition of particular epigenetic signatures, when applied to the precision medicine context of lung cancer, should be carried out in an easier and comprehensive way [10,11].

Similarly to the Tumor Mutation Burden (TMB), one of the emerging biomarkers in cancer that is gaining attraction is the definition of a Tumor Methylation Burden (TMeB). This should be considered a dynamic metric simultaneously reflecting the density and level of DNA methylation changes in multiple cancer-related genes or/and regions at a single “CpG” resolution. The TMeB assessment not only could be fundamental to discriminating tumor-derived from non-tumoral samples or monitoring clonal evolution during tumor recurrence, but it could also help to identify and profile different histological and clinical cancer subgroups with high sensitivity and specificity [12,13]. In this regard, establishing a more robust and integrated algorithm that includes DNA methylation, immune and expression profile and genomic signatures, as well as copy number variations (CNVs), microsatellite instability, and the TMB, could also significantly improve the accuracy of prognostic predictions of lung cancer patients’ survival at any stage of disease [14,15,16].

In recent decades, many research groups highlighted, using extensive and relevant data, the emerging role of epigenetic biomarkers as promising tools for cancer diagnosis [17,18]. The obtained epigenetic models, in combination with current diagnostic protocols, will improve the early diagnosis and outcome of lung cancer patients, including those at an advanced stage and undergoing different treatments, as immune checkpoint inhibitor (ICI) therapies [19].

Recently, Guidry and colleagues defined a DNA methylation age (DNAm age) pattern with a complete characterization of the clinical and genomic profiles of lung resected adenocarcinoma (LUAD) tumors. Unsupervised DNA methylation clustering identified six molecular subgroups of LUAD tumors with distinct clinical and microenvironmental characteristics. Using both epigenetic and immunological signatures, the detection of promising predictors of LUAD early-stage patients paves the way fort he possible tailoring of targeted therapy or immunotherapy-based approaches after surgical resection in this subgroup of lung cancer patients [20].

The studies of Cai and collaborators described a strong link between the methylome alterations and TMB degree in naïve LUAD or lung squamous cell carcinoma (LUSC) Chinese patients. Methylome-wide analysis revealed widespread changes in lung cancer-associated DNA methylation patterns among >800,000 CpG sites covering key features of the human whole genome, particularly in high-TMB cancer tissues and CNVs settings [21]. A growing body of research activities is focusing on this field and increasingly interesting evidence is emerging, thus corroborating the link between small-group/single-gene or genome-wide DNA methylation patterns and the prediction of response to ICI treatments [22,23].

In a multicenter, retrospective analysis, a DNA methylation profile was assessed to determine the efficacy of anti-Programmed Cell Death Protein 1 (PD-1) treatment in patients with stage IV NSCLC. The epigenomic profile was based on a microarray DNA methylation signature (EPIMMUNE) in a discovery set of tumor samples from 142 patients treated with nivolumab or pembrolizumab (34 in the discovery cohort, 47 in the EPIMMUNE validation cohort, and 61 in the derived methylation marker cohort). An intriguing result came from the EPIMMUNE signature (forkhead box P1, *FOXP1* status), since this was significantly associated with progression-free survival (PFS) and overall survival (OS), suggesting that EPIMMUNE may be a good predictor of anti-PD-1-ICI response [24].

Similarly, an association of specific CpG sites with the response to anti-PD-1/PD-1 agents in advanced/metastatic NSCLC patients was reported by Kim et al. In this study, tumor samples obtained from NSCLC patients underwent Illumina 850K/EPIC platform analysis. DNA methylation changes were ascertained between responders and non-responder patients for more than 300 differentially hyper/hypo methylated CpG sites. The obtained LASSO regression risk model of eight specific genes linked to treatment response suggests that methylation patterns should be restricted to a small group of genes in specific lung cancer patients that are potentially representative of a TMeB, thus supporting the definition of a complex molecular determinants network for improving the efficacy of immune checkpoint inhibitors [25].

Additionally, TMeB variations and the tumor microenvironment are involved in resistance to anti-tumor responses, e.g., through activating Epidermal Growth Factor Receptor (*EGFR*) mutations in tyrosine kinase inhibitors (TKIs) in NSCLC patients [6,26]. As lung cancer grows via clonal evolution, dynamic molecular changes are acquired, generating subclonal variation, which can be defined as intra-tumoral heterogeneity (ITH) [27].

The differential spread of methylation at CpG sites, from invasive lung adenocarcinoma to its precursors, atypical adenomatous hyperplasia, adenocarcinoma in situ, and minimally invasive adenocarcinoma, demonstrates that, similarly to gene mutations, methylation profiles also alter during tumor evolution. Later-stage lesions showed a markedly greater level of methylation ITH and a progressive increase in methylation abnormalities. Parallel methylation and genetic evolution are suggested by the phylogenetic patterns derived from methylation aberrations and those based on somatic mutations [28].

The possible influence of methylation on chromosomal instability, mutagenesis, and the tumor immune microenvironment during the early carcinogenesis of lung adenocarcinomas is further highlighted by the fact that higher global hypomethylation is linked to both higher mutation and an increase in the copy number variation burden [28].

Finally, the latest frontiers of methylation profiling suggest that, as for tumor tissues, circulating tumor DNA (ctDNA) could represent a useful matrix to measure methylation-level variations and perform a non-invasive disease monitoring through liquid biopsies, as well as supporting lung cancer subtyping in a minimally invasive procedure to complement standard diagnostic procedures [29,30].

In summary, the TMeB represents a burgeoning frontier in lung cancer management in the era of precision medicine. Based on the latest scientific advances, clarifying and translating the TMeB into clinical practice, starting from an analysis of the different biological matrices, could significantly improve the outcome of lung cancer patients, moving us closer to truly personalized medicine.

In lung cancer systems’ dynamic biology, embracing complexity to develop better anticancer therapeutic strategies is the key, and the concept of epigenetics appears both exciting and overwhelming and could prove to be a game-changer.
